# Successful pregnancy in the blind hemicavity of Robert’s uterus: a rare case report and brief literature review

**DOI:** 10.1186/s12884-023-05541-5

**Published:** 2023-03-28

**Authors:** Lingling Dong, Shi Qin, Ronghua Che, Jindan Pei, Xiaolin Hua

**Affiliations:** Department of Obstetrics, Shanghai First Maternity and Infant Hospital, Shanghai Tongji University School of Medicine, 2699 West Gaoke Road, Shanghai, 201204 China

**Keywords:** Robert’s uterus, Blind hemicavity, Successful pregnancy, Case report

## Abstract

**Background:**

Robert’s uterus is a rare congenital anomaly, characterized as an asymmetric septate uterus that has a blind hemicavity with unilateral menstrual fluid retention and a unicornuate hemicavity connecting to the cervix unimpededly. Patients with Robert’s uterus generally present with menstrual disorders and dysmenorrhea, and some may have reproductive problems as well, including infertility, recurrent miscarriage, preterm labor and obstetric complications. In this case, we describe a successful pregnancy implanted on the obstructed hemicavity and delivered a liveborn girl. Meanwhile, we highlight diagnostic and therapeutic difficulties in patients with atypical symptoms of Robert’s uterus.

**Case presentation:**

A 30-year-old Chinese primigravida sought for emergency treatment at 26 weeks and 2 days of gestation because of preterm premature rupture of membranes (PPROM). At the age of 19, the patient was misdiagnosed with hyperprolactinemia and pituitary microadenoma for showing symptom of hypomenorrhea and was suspected to have a uterine septum in the first trimester. She was diagnosed with Robert’s uterus at 22 weeks of gestation by repetitious prenatal transvaginal ultrasonography, which was subsequently confirmed by magnetic resonance imaging. At 26 weeks and 3 days of gestation, the patient was suspected to have oligohydramnion, irregular uterine contraction, and umbilical cord prolapse, and she expressed a strong will of saving the baby. Emergency cesarean delivery was performed and a small hole, together with several weak spots, was found at the lower and back wall of the septum of the patient. The treatment was effective and both the mother and the infant, who had an extremely low birth weight, were discharged in good health conditions.

**Conclusions:**

Pregnancy in the blind cavity of Robert's uterus with living neonates is incredibly rare. In our case, the favorable outcome may result from the unusual hole found at the septum, which may play a role in communicating amniotic fluid between the two hemicavities so to keep the neonate alive. we highlight the importance of early diagnosis and pre-pregnancy treatment of this uterine malformation, and the timely termination of pregnancy, for improving birth quality and reducing mortality.

## Background

Robert's uterus is a rare abnormality of Müllerian duct with only a few reported cases. It was initially proposed by Héléne Robert and described as an asymmetrical forked uterus with one-sided menstrual retention [1]. Robert's uterus is an asymmetrical variation of the uterine septum, characterized by a complete longitudinal partition asymmetrically separating the uterine cavity from the fundus of uterus to the internal cervical ostium, resulting in an inaccessible uterine hemicavity and a unicornuate uterine hemicavity with a general external silhouette [2]. The lack of communication between the two uterine hemicavities contributes to the retention and reflux of menstrual blood, leading to hematometra, ipsilateral hematosalpinx, intense dysmenorrhea, peritoneal implantation of endometriosis, and secondary reproductive dysfunctions, such as infertility, miscarriage, preterm labor and malpresentation-caused obstructed labor [3, 4]. Some patients were misdiagnosed due to the lack of typical clinical manifestations or accurate imaging examination. For example, it has been reported that a patient received two unnecessary surgeries because of misdiagnosis and only after the diagnosis of Robert’s uterus and the subsequent consiappropriate management was her cyclical abdominal pain relieved [4]. Up to now, minimally invasive ultrasound-guided hysteroscopic metroplasty is considered the best way to correct this malformation as it has the potential to normalize the uterine morphology and function [2]. There is no shortage of successful cases in which the patients were able to conceive naturally after surgery and deliver healthy newborns[5], indicating early diagnosis and timely remodeling are essential for cases where infertility is an issue.

We report the first case of pregnancy located in the blind hemicavity of Robert’s uterus without pre-pregnancy surgery. The patient conceived naturally and had atypical clinical manifestations resulted from the unique malformation of the uterine septum, reprensenting a unique and informative case. We emphasize the special type of this rare disease and provide clinical inspirations for early diagnosis and treatment of Robert’s uterus.

## Case presentation

A 30-year-old pregnant woman, gravida 1, para 0, was admitted from emergency room for premature rupture of membranes (PROM) at 26 weeks and 2 days of her gestation. She established menarche at the age of 13 and has had regular menstrual cycles at a 30-day interval and a duration of 2–3 days since then. Despite of scrimpy menses, she had no dysmenorrhea since its onset. The patient was considered hyperprolactinemia and pituitary microadenoma due to hypomenorrhea after a brain MRI scanning conducted at a local hospital. The patient was treated with endocrine drugs for years but there was no substantial improvement in her period.

**Fig. 1:**
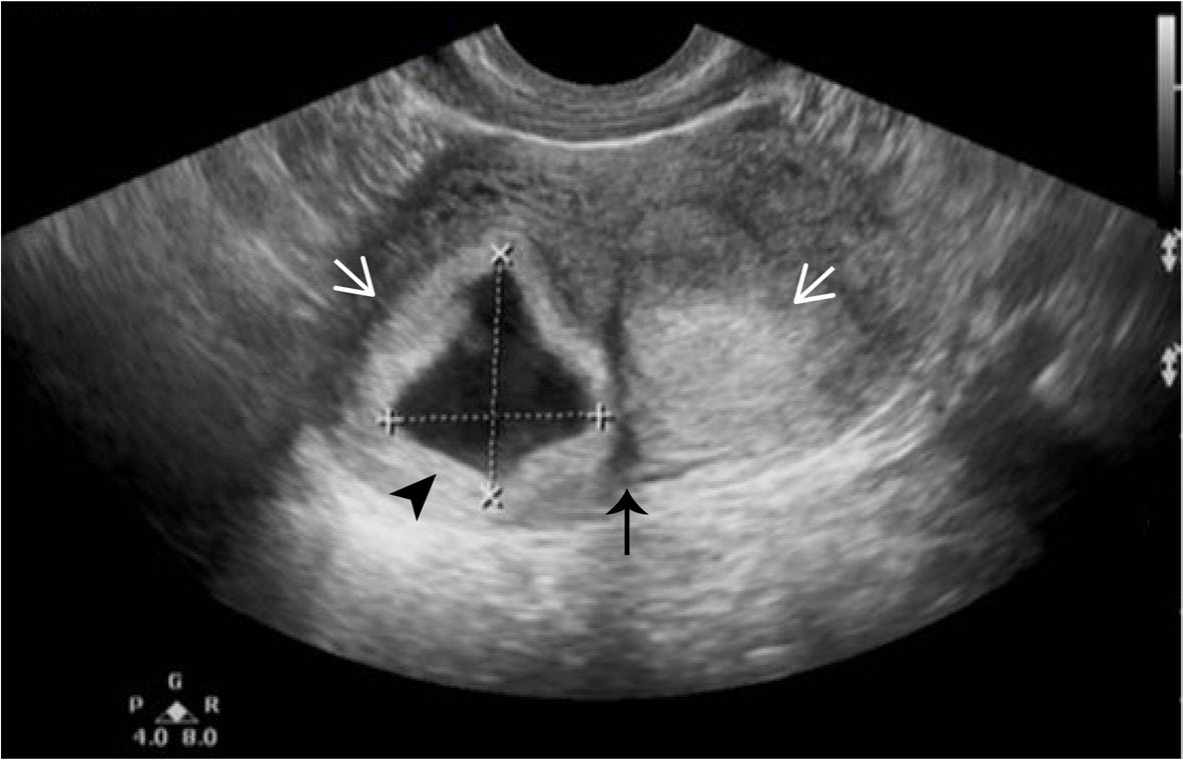
Two-dimensional ultrasound images during the first trimester. A uterine septum (black arrow) divides the uterus into two hemicavities (white arrows). Pregnancy occurs in the right blind hemicavity (black arrow head)

**Fig. 2:**
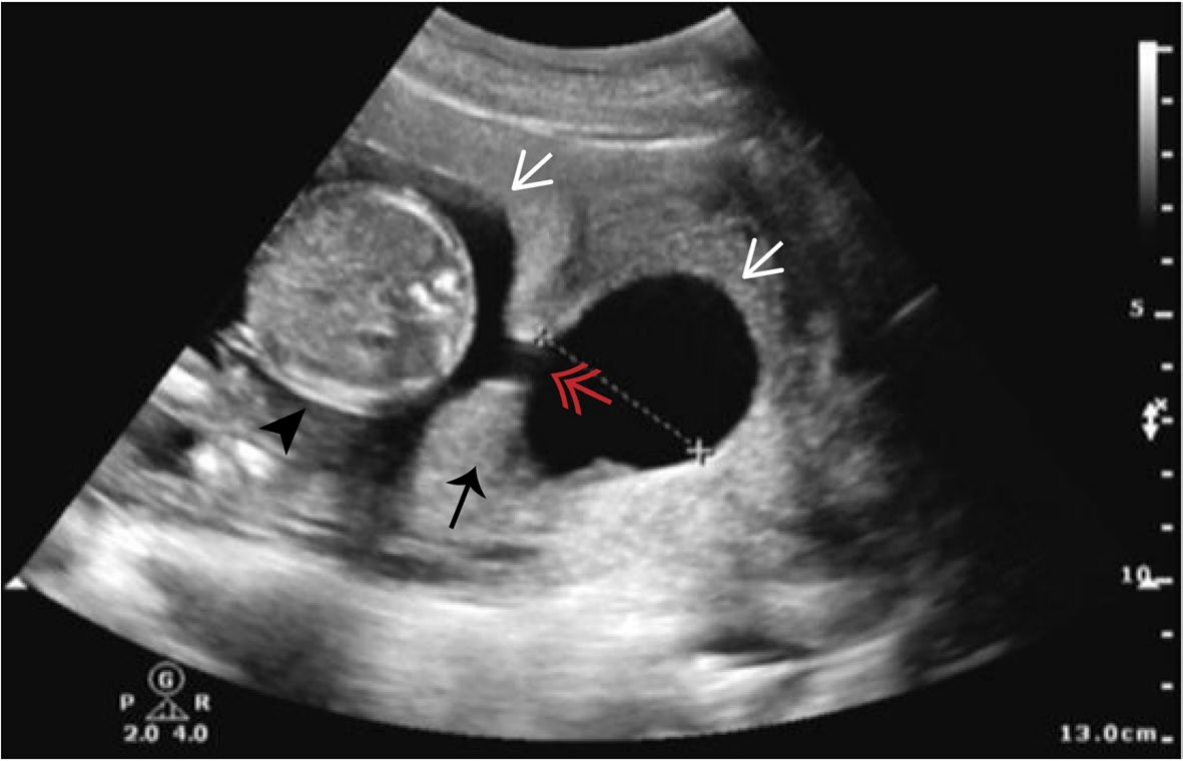
Ultrasonography for diagnosing fetal malformations. The two hemicavities (white arrows) are connected by a 10 mm hole in diameter (red double arrow) in the uterine septum (black arrow). Pregnancy occurs in the right blind hemicavity (black arrow head)

**Fig. 3:**
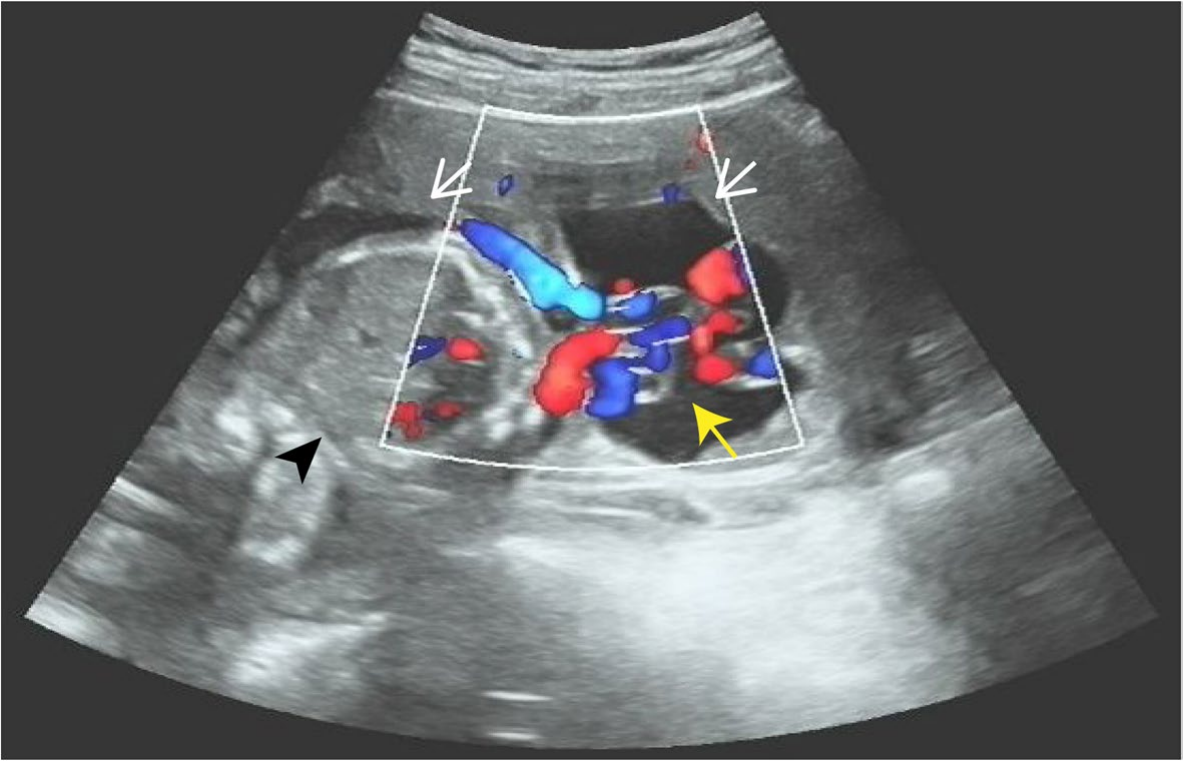
Ultrasonography images show the amniotic sac stretches across two hemicavities (white arrows). The fetus (black arrow head) is located in the right hemicavity with little amniotic fluid, whereas the umbilical cord (yellow arrow) extends to the contralateral hemicavity with most amniotic fluid

**Fig. 4:**
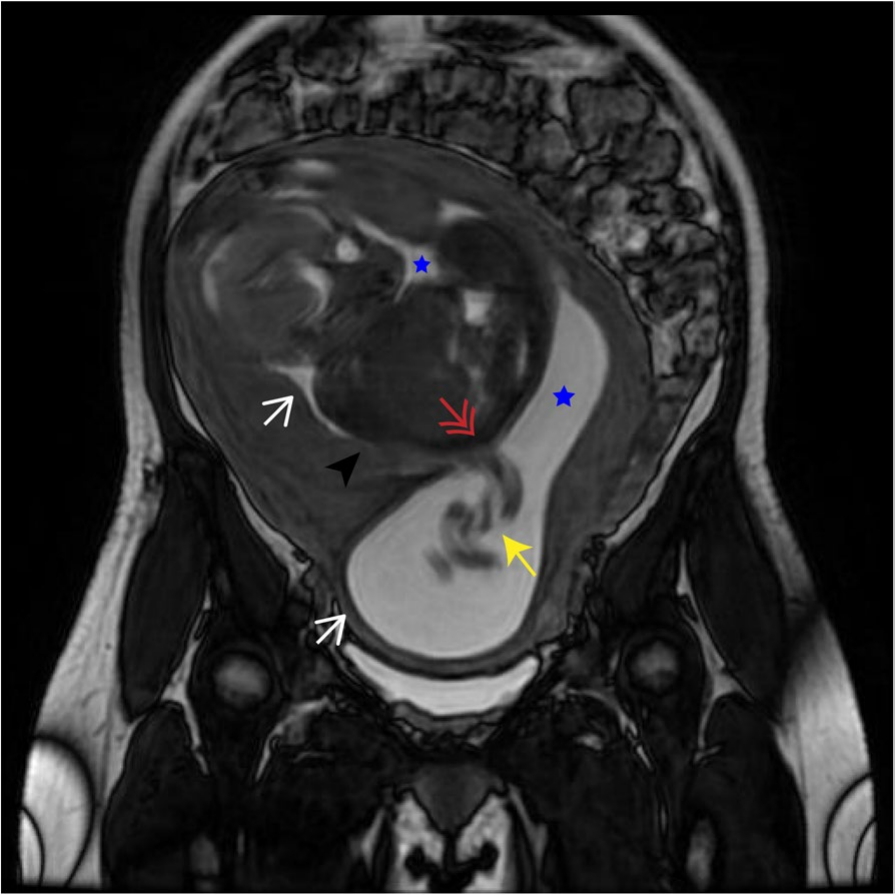
Magnetic resonance images. The amniotic sac lying across two hemicavities (white arrows) by a 10 mm defect (red double arrow) at the septum. The fetus (black arrow head) with little amniotic fluid (blue pentagram) in the right hemicavity and the umbilical cord (yellow arrow) with most amniotic fluid in the left hemicavity

This is a natural pregnancy and the patient visited our hospital periodically for prenatal check-ups. The first obstetric ultrasonography at 7 weeks of gestation showed splayed endometrium and an intrauterine septal echo, indicating the probability of a dysmorphic uterus with the pregnancy sac and embryo in the right uterine hemicavity (Fig. 1). The subsequent NT ultrasonography at 12 weeks of gestation suggested low risk of fetal chromosomal abnormalities, and all the other pre-pregnancy examinations were normal. During the middle pregnancy, at 22 weeks of gestation, the patient did an ultrasound screening for fetal macrostructural malformation. The result showed an intrauterine septum dividing the uterine cavity into two asymmetric hemicavities, indicative of Robert’s uterus. The result also indicated oligohydramnion in the right hemicavity with a depth of 16 mm, and fetal growth restriction at 3.5 percentile of normal weight at the same age due to the rare pregnancy in the uterine hemicavity (Fig. 2). We performed confirmatory transvaginal ultrasound (Fig. 3) and pelvic MRI (Fig. 4) to verify that the fetus was confined to the right blind hemicavity by the septum, surrounded by little amniotic fluid, whereas the umbilical cord was floated in the contralateral hemicavity with quadruple amniotic fluid, achieved by a 10 mm-wide hole at the septum.

**Fig. 5:**
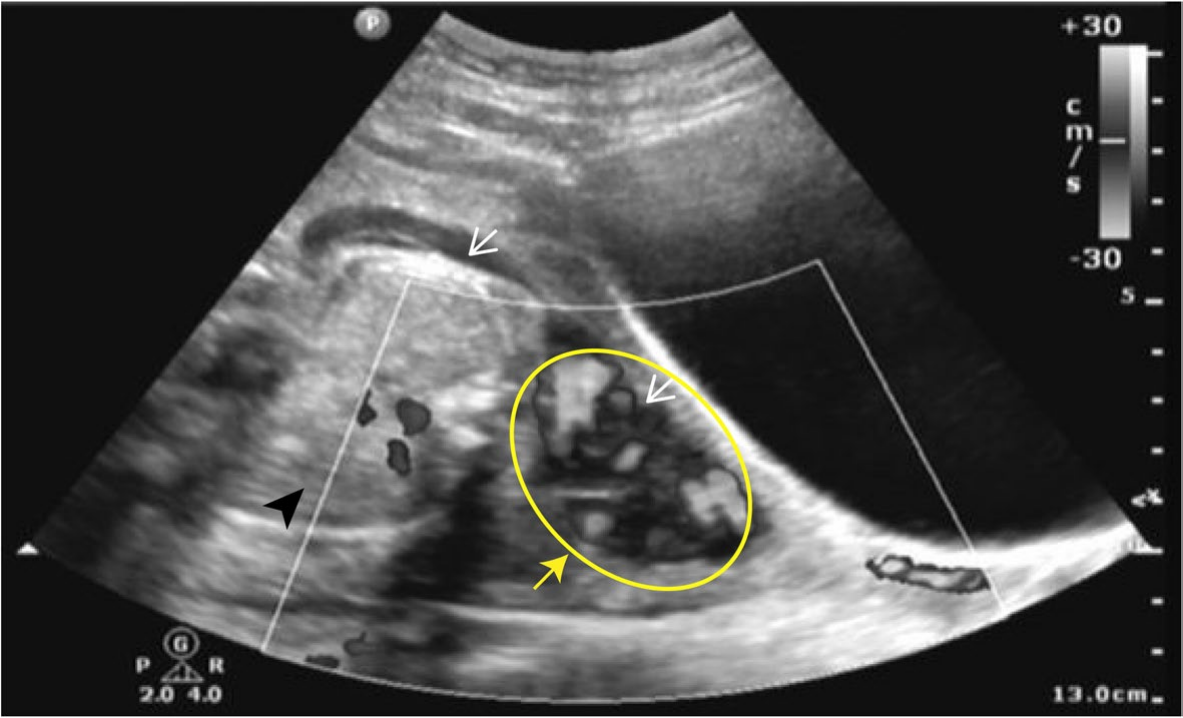
Obstetric ultrasonography after admission. There is hardly any amniotic fluid in the amniotic sac in both hemi-cavities (white arrows). The fetus curls up in the right hemicavity and the umbilical cord (yellow circle and yellow arrow) is huddled in the left side directly above the internal cervical os

The occurrence of PPROM at 26 weeks and 2 days of the patient’s gestation has no obvious incentive with minimal vagina bleeding, slight abdominal pain, and no bloating or vomiting. The vulva, vagina and cervix were anatomically normal by gynecological examination, and so was the development of the gravid uterus. Considering the outflow of amniotic fluid and grade II polluted amniotic fluid, she was reevaluated by an obstetric ultrasonography after admission. According to the ultrasonography, there was hardly any amniotic fluid in the amniotic sac and the fetus was presented in a transverse position in the right hemicavity. More alarmingly, we found the umbilical cord was huddled in the left hemicavity directly above the internal cervical os (Fig. 5). The next day, at 26 weeks and 3 days of gestation, the patient had irregular contractions. Despite temporary normal inflammatory indicators, we took into account of the drained amniotic fluid, the piled-up umbilical cord above the cervix opening and the contraction, and suggested an immediate termination of pregnancy in order to avoid severe complications such as umbilical cord prolapse, intrauterine infection, and even maternal death. The patient and her families were informed of the developmental immaturity of the very preterm infant, great rescue expenses and a probable failure of rescue, but they still expressed a strong wish to save their baby. We sought for the pediatric consultation and confirmed the medical care capacity to support the rescue of the premature at this gestational age. Thus, after fetal lung maturation with dexamethasone and cranial nerve protection with magnesium sulfate injection, an emergency cesarean was performed. During the laparotomy, we found the uterus was deviated to the right side with a normal external shape. Into the uterine cavity, the fetus was located in the right hemicavity in the transverse position. We delivered the fetus rapidly, and found the placenta was mainly implanted on the fundus of the right side of the uterus, while a small part of the placenta was located in the septum. After removing the placenta manually, we confirmed a longitudinal septum which extended from the fundus to the middle-lower uterus. It was about 10 mm thick near the anterior uterine wall and presented as a membrane at the rear, dividing the uterine cavity into two asymmetric hemicavities. With the delivery of the infant, the septum was squashed against the left uterine wall. Considering the membrane-like tissue was completely ruptured at the weak spots during delivery, our intraoperative gynecological evaluation came to the decision of leaving the asymmetrical uterine septum alone and we suggested postoperative follow-ups. Postpartum ultrasonography did not find any significant abnormal echo in the uterine cavity, and the mother was discharged three days later. The extremely premature infant scored 4–7 for the 1–5 min newborn Apgar test and was transferred to an authoritative pediatric hospital for further treatment. After 128 days of positive rescue and hospitalization, the baby girl was discharged with all the indicators of body health being normal, which is indeed a great gratification.

## Discussion

Approximately 5.5% women in the general population have uterine anomalies [6]. These malformations always come with imperceptible symptoms, impairing female reproductive functions undetectably [7]. According to the European Society for Gynecological Endoscopy (ESHRE/ESGE) classification, Robert’s uterus is an extraordinary variant of septate uterus categorized to class U2b modalities, and only a few clinical cases have been reported [8]. Patients with Robert’s uterus generally show classical complications, including hematometra, pelvic pain, intense dysmenorrhea, endometriosis and infertility [9–11]. Dividing into two asymmetric hemicavities by an intrauterine septum, the right horn is more likely to be obstructed. It perhaps due to the slightly advanced development of the left Müllerian duct than the right one. However, recent publications have reported atypical clinical manifestations of a few special variants of Robert’s uterus: some patients have no hematometra and dysmenorrhea histories [2], some have ipsilateral urinary anomalies [12], and some are presented with left blind hemicavities [13].

Unlike patients with presentations in above-mentioned reports, our patient had few related symptoms and conceived natrually. The natural pregnancy in the blind hemicavity and the absence of hematometra, hematosalpinx and dysmenorrhea clearly demonstrated that the endometrium was functional and the menstrual blood was eliminated in a unique way. The repeatable ultrasound and pelvic MRI provided us clues and evidence for this rare variant of Robert’s uterus. The imaging confirmed a 10 mm-wide hole at the septum, through which the menstrual blood, the sperm or the zygote could be transferred. Thus, we supposed that the ovum from both sides could be fertilized through the small hole, and the embryo was implanted on the right hemicavity eventually. This could also explain the fetus locating in the right hemicavity and the umbilical cord floating in the left hemicavity: the intact amniotic cavity was transverse across the two hemicavities, with the folded umbilical cord stuck in the hole. In addition, we attributed fetal growth retardation and PPROM to the pregnancy in the untreated Robert’s uterus. As mentioned above, there was limited growing space and few amniotic fluid in the blind hemicavity. We believed that the one-fifth of amniotic fluid surrounding the fetus could not provide adequate placental reserve capacity, meet the blood and oxygen requirement, or allow musculoskeletal stretching of the fetus, resulting in the fetal growth retardation. Apparently, the right blind hemicavity could afford neither the growth of the fetus nor the accumulation of the amniotic fluid, so the risk of the occurrence of PROM might increase with the gestational age.

As for the timing of termination of pregnancy, we believed that inflammatory indicators, the location of the umbilical cord, the fetal heart rate monitoring, characteristics of the amniotic fluid, and patients’ wills should be taken into account. Clinicians should avoid intrauterine infections, umbilical cord prolapse or other complications. In our case, the umbilical cord was directly piled up above the internal cervical os because of the massive loss of amniotic fluid. It particularly concerned us that the umbilical cord would prolapse with the uterine contraction. Moreover, we suggest monitoring fetuses at over 28 weeks for a good reflection of the fetal heart rate and contraction. Our fetus was too immature to be monitored because of her underdeveloped sympathetic and parasympathetic nervous systems. Fortunately, despite the very premature birth, the patient and the infant were discharged with no complications successively, and we suggested gynecological follow-ups for her Robert’s uterus.

To our best knowledge, this is the first case to report an asymptomatic Robert’s uterus and a natural pregnancy in the blind hemicavity with favorable outcomes. In our study, the Robert’s uterus was ignored due to atypical manifestations and complications of endocrine-related disorders before pregnancy. This case reminds us to be alert to patients with special types of uterine anomalies. Although our patient did not undergo intraoperative metroplasty because of the rupture of septum during delivery, we encourage accurate diagnosis and early treatment for Robert’s uterus in further clinical practice, which would not only relieve patients’ discomfort but also reduce pregnancy complications.

## Conclusion

This case highlights the diagnostic and therapeutic difficulties with patients having atypical symptoms of Robert’s uterus. Clinicians need to take uterine anomalies into account for patients with irregular menstruations. It also inspires us the importance of early diagnosis and timely termination of pregnancy under this circumstance for improving birth quality and reducing mortality. The fetus in the blind hemicavity could be saved if he/she develops well.

## Data Availability

All data generated or analyzed during this study are included in this published article.

## Abbreviations

PROM: Premature rupture of membranes
PPROM: Preterm premature rupture of membranes
